# Agreement-Based Validation of ISOMETRO for Upper-Limb Isometric Tension Measurements

**DOI:** 10.3390/s26051504

**Published:** 2026-02-27

**Authors:** José Luis González-Montesinos, Jorge del Rosario Fernández-Santos, David Jiménez-Pavón, Alejandro Sánchez-Delgado, Rubén Aragón-Martín, Juan Manuel Escudier-Vázquez, Vanesa España-Romero

**Affiliations:** 1 Department of Physical Education, Faculty of Education Sciences, University of Cádiz, 11519 Cádiz, Spain; jgmontesinos@uca.es; 2 Biomedical Research and Innovation Institute of Cádiz (INiBICA) Research Unit, 11009 Cádiz, Spain; jorgedelrosario.fernandez@uca.es (J.d.R.F.-S.); david.jimenez@uca.es (D.J.-P.); juanmanuel.escudier@uca.es (J.M.E.-V.); vanesa.espana@uca.es (V.E.-R.); 3 GALENO Research Group and Department of Physical Education, Faculty of Education Sciences, University of Cádiz, 11519 Cádiz, Spain; 4 MOVE-IT Research Group and Department of Physical Education, Faculty of Education Sciences, University of Cádiz, 11519 Cádiz, Spain; 5 CIBER of Frailty and Healthy Aging (CIBERFES), 28029 Madrid, Spain; 6 Computational Social Science DataLab (CS2 DataLab), University Research Institute for Sustainable Social Development (INDESS), University of Cádiz, 11405 Jerez de la Frontera, Spain; alejandro.sanchezdelgado@uca.es; 7 C-HIPPER Climbing Research Association, 11100 Cádiz, Spain

**Keywords:** isometric force, isometric strength, agreement analysis, criterion agreement, force plate, load cell, measurement chain, muscular strength, exercise testing, biomechanics, biomedical sensors

## Abstract

**Highlights:**

**What are the main findings?**

- ISOMETRO demonstrated excellent agreement with an independent force-plate reference for upper-limb isometric tensile force under a standardized vertical laboratory configuration.
- Bland–Altman analysis, concordance metrics (CCC/ICC), and mixed-effects modeling showed near-unity agreement, negligible systematic bias, narrow limits of agreement, and confirmed internal measurement-chain consistency.

**What are the implications of the main findings?**

- Under controlled vertical alignment, ISOMETRO provides peak-force measurements that closely match an independent criterion reference system.
- The guided-rail architecture supports standardized laboratory-grade upper-limb tensile testing, although further studies are required to establish reliability and validate additional force directions and real-world applications.

**Abstract:**

Muscular fitness is a key component of health and athletic performance, and isometric strength is a widely used indicator. This study reports an agreement-based validation of the Isometric Strength Measurement Device (ISOMETRO) for upper-limb isometric tension measurements under controlled laboratory conditions. Twenty-one healthy young amateur rock climbers (11 men and 10 women) performed four upper-limb tensile tests (shoulder adduction at 90°, shoulder adduction at 60°, shoulder extension at 90°, and elbow extension at 90°). Agreement with an independent criterion device was evaluated using a force plate, while a series-connected load cell was used as an internal consistency check of the measurement chain. Linear mixed-effects models showed that ISOMETRO strongly predicted force plate values (β = 0.999, SE = 0.002, *p* < 0.001), with a marginal R^2^ > 0.99. Bland–Altman analysis indicated negligible bias (−0.08 N) and narrow limits of agreement (−4.97 to 4.81 N), and concordance was excellent (CCC ≥ 0.996). The series-connected load cell comparison also showed near-unity agreement (β = 0.998, SE = 0.003, *p* < 0.001), supporting internal measurement chain integrity. These findings support excellent agreement between ISOMETRO and force plate measurements for upper-limb tensile isometric testing along the vertical axis in young amateur rock climbers under controlled laboratory conditions. However, given the specific sample characteristics and the strictly vertical laboratory configuration, these results should not be generalized to other populations, joint angles, force directions, or non-laboratory environments without further validation. Further studies are needed to confirm performance in more diverse contexts and to establish reliability for repeated-measurement applications.

## 1. Introduction

Muscular fitness is a key component of physical fitness and has been consistently linked to health and performance outcomes [1,2,3]. Operationally, muscular fitness reflects the capacity of skeletal muscles to generate force, sustain contractions, and resist fatigue [2]. Contemporary perspectives emphasize that muscular fitness supports health, rehabilitation, and sport performance along a continuum, making valid and reproducible strength assessment relevant across diverse physiological and functional contexts. In addition, recent biomechanical investigations have highlighted how precise quantification of force production and joint loading patterns can inform injury prevention strategies and optimize movement execution in dynamic tasks [4], further underscoring the broader relevance of accurate force measurement systems in human performance research.

Isometric strength (IS) testing is widely used because it can be implemented efficiently and provides clinically and functionally meaningful information. For example, handgrip IS has been associated with mortality risk and functional independence [5], and quadriceps IS has been linked to chronic conditions such as coronary artery disease [6], chronic obstructive pulmonary disease [7], and knee osteoarthritis [8,9]. In rehabilitation, IS assessment is frequently used to characterize normal versus pathological patterns [8], including tendinopathy [10,11] and immobilization-related muscle atrophy [12]. From a performance standpoint, IS and isometric training have shown relevance in multiple sports, including climbing [13], sailing [14], judo [15], tennis [16], football [17], basketball [18], and Paralympic disciplines [19].

Multiple tools are available to assess muscular strength, each with practical and methodological constraints. Isokinetic dynamometry provides precise torque measurements under controlled velocities [20,21,22,23], but its high cost, limited portability, and requirement for specialized infrastructure restrict routine implementation [20,24]. Alternatives include hand-held dynamometers [21,25], back dynamometers [26], force plates [27], and stand-alone load cells. Hand-held dynamometers can be accessible but are sensitive to examiner strength, positioning variability, and inter-rater inconsistency [28]. Back dynamometers are typically limited to trunk-dominant assessments and offer less flexibility for joint-specific testing [29]. Force plates are highly reliable for measuring ground reaction forces, yet they are most commonly applied to lower-limb or whole-body tasks and may be less suited to isolated upper-limb traction tests unless the mechanical setup ensures an interpretable relationship between traction and ground reaction forces [30]. Stand-alone load cells often require external anchoring or stabilization, which can introduce between-trial variability and reduce reproducibility across sessions and operators [31].

Recent portable and sensor-based technologies—including smartphone-based force sensors [32], portable traction dynamometers [33], and wearable systems for estimating joint torque [26]—have improved accessibility. In parallel, emerging analytical approaches such as self-supervised learning applied to sensor-derived biomechanical signals have expanded the capacity to extract meaningful performance and event-detection metrics from wearable inertial data in sports and human performance contexts [34]. However, challenges remain regarding standardization, calibration stability, and criterion agreement when compared with reference devices such as force plates or industrial-grade load cells, particularly for standardized upper-limb tensile testing [35,36]. Consequently, there is a practical need for systems that facilitate consistent alignment and repeatable testing conditions while maintaining strong agreement with reference measurements under well-defined configurations.

To address these limitations, the Isometric Strength Measurement Device (ISOMETRO) was developed as a wall-mounted system designed to measure upper-limb isometric tension under a vertically aligned traction configuration. ISOMETRO incorporates a guided vertical rail and an adjustable sled intended to support consistent alignment and reduce reliance on external stabilization. From a practical standpoint, the system occupies limited space (2000 × 80 × 80 mm), weighs 17.6 kg, and can be installed in approximately 60 min. These design features aim to facilitate standardized upper-limb tensile assessments under controlled conditions.

Accordingly, the purpose of this study was to conduct an agreement-based validation of ISOMETRO for upper-limb isometric tension measurements during four standardized tensile tests performed by young adults under controlled laboratory conditions. Independent concurrent validity was evaluated using a force plate as the criterion device, while a series-connected load cell was used as an internal consistency check of the measurement chain. We hypothesized that ISOMETRO would demonstrate excellent agreement with force plate measurements for the vertical component of upper-limb tensile isometric force under the specific testing configuration used in this study.

## 2. Materials and Methods

### 2.1. Participants

Twenty-one healthy young adults volunteered to participate in a laboratory-based experimental study conducted within the framework of the High-Performance International Rock-Climbing Research Group (C-HIPPER) (11 men and 10 women; age: 20.4 ± 2.8 years, height: 173 ± 8.1 cm, weight: 69.6 ± 11.1 kg).

All participants were amateur rock climbers whose primary sport over the previous five years was climbing. They regularly practiced both indoor (climbing wall) and outdoor rock climbing and reported engaging in at least three climbing sessions per week (≥150–180 min/week), typically performed at moderate-to-vigorous intensity. Some participants occasionally combined climbing with other forms of physical activity; however, climbing represented their main sport practice during this period. None were classified as elite athletes, defined as individuals competing at national or international levels or holding a professional or semi-professional sport license. All participants were free of any musculoskeletal or neurological disorders that could affect upper-limb strength.

Participants were instructed to refrain from intense exercise for at least 48 h before testing and to avoid food intake for two hours prior to the evaluation.

Before participation, all individuals were informed about the purpose, procedures, and potential risks associated with the study and provided written informed consent. The study was conducted in accordance with the Declaration of Helsinki and approved by the University Ethics Committee (Reference: 0229-N-19). All data were collected within the C-HIPPER research framework.

### 2.2. Sample Size Estimation

An a priori sample size estimation was performed based on previous validation studies of isometric strength measurement devices, which consistently report very high associations between criterion and validated instruments (typically r ≥ 0.90–0.95) [26,27,33,37]. Using a conservative expected correlation of r = 0.90–0.95 and an alpha level of 0.05, standard power calculations indicate that a minimum of approximately 12–15 participants is generally sufficient to detect such associations with adequate statistical power in validation contexts.

Based on these considerations, a sample of 21 participants was selected to ensure stable estimation of agreement metrics and to provide robustness against potential data loss or unusable trials. Although multiple measurements were obtained from each participant across tests and sides, all statistical analyses accounted for the clustered structure of the data using mixed-effects modeling, with participants treated as random effects.

The selected sample size is consistent with previously published device-validation studies in this field, which typically include between 15 and 50 participants [26,27,33,37], and is considered appropriate for an agreement-based validation conducted under controlled laboratory conditions.

### 2.3. Materials

Three measurement systems were used simultaneously to record isometric force (IF) during all tests: the ISOMETRO device (Cádiz, Spain), an external load cell (MuscleLab–Ergotest, Langesund, Norway), and a force plate system (MuscleLab–Ergotest, Langesund, Norway).

(a) ISOMETRO

ISOMETRO is a patented device developed by the University of Cádiz (OEPM No. ES-2646730_B2) [38] and designed to quantify isometric force under controlled mechanical alignment. In the present study, the device was configured exclusively for vertical tensile measurements.

**Figure 1:**
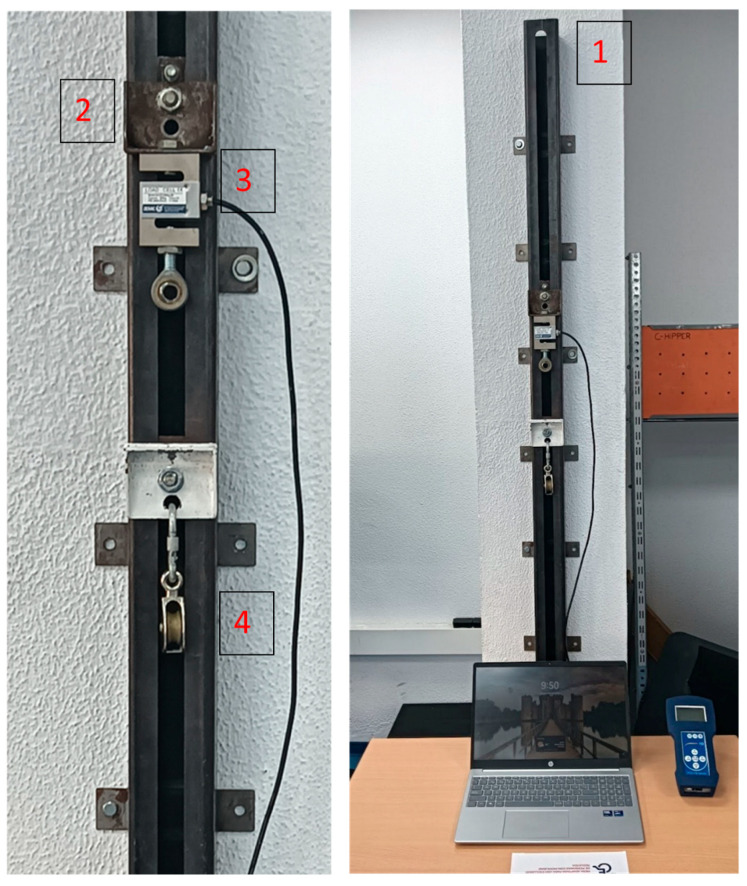
ISOMETRO system (University of Cádiz). Main components: (1) rail; (2) sled; (3) load cell; (4) optional transmission pulley for alternative force directions.

**Figure 2:**
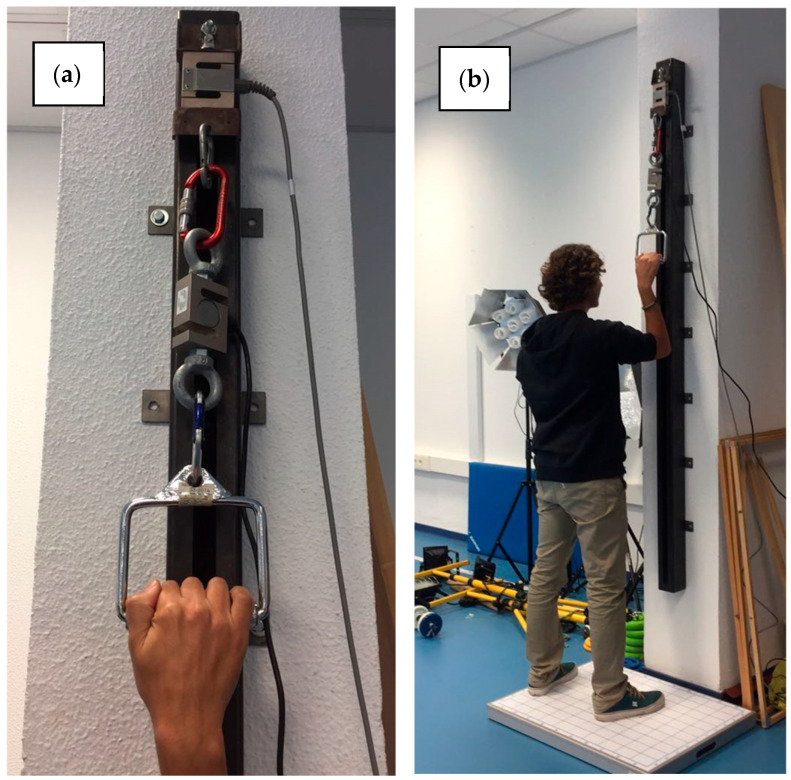
(**a**) External load cell connected in series with the ISOMETRO system; (**b**) participant positioning on the force plate during testing. ISOMETRO, load cell, and force plate recorded force simultaneously during all tests.

The system consists of a vertically mounted guide rail (2000 × 80 × 80 mm), a sliding sled (80 × 80 × 35 mm), an integrated load cell, and an optional pulley assembly (Figure 1 and Figure 2). The rail was installed using a commercially available electronic level. The guidance mechanism mechanically constrains all translational and rotational motion except vertical displacement (*Z*-axis), allowing movement only along this axis with an off-axis tolerance of ±0.5°. Installation of the guiding rail, sled, and load cell requires approximately 60 min.

The load cell integrated into ISOMETRO is an S-type sensor (PCE-FB 2K, PCE Instruments, Albacete, Spain), manufactured in nickel-plated alloy steel with IP67 protection. It has a measurement accuracy of ±0.1% full scale (±1.0 N), a maximum load capacity of 3000 N, and a combined error ≤ ±0.020. Operating temperature range is −35 °C to +65 °C.

In the present configuration, all tests were performed strictly along the vertical axis; therefore, the pulley system was not used and remained static throughout testing. Consequently, no dynamic frictional effects or transmission efficiency corrections were required. Potential frictional effects associated with pulley redirection were not evaluated in this study and should be addressed in future investigations involving non-vertical or multi-planar loading configurations.

The sliding sled supports the load cell, which is rigidly screw-mounted to the carriage. A carabiner attached to the lower end of the load cell transmits tensile force during testing. The vertical adjustability of the sled allows precise alignment with participant anthropometry and joint positioning, ensuring standardized traction angles across participants and test conditions.

The analogue signal generated by ISOMETRO is digitized using a 24-bit analogue-to-digital converter integrated into the system microcontroller. Force–time data were sampled at 200 Hz. No filtering or smoothing was applied; all analyses were performed on raw signals, and peak force was extracted directly from the unprocessed trace.

The ISOMETRO load cell is factory calibrated (Certificate NTEP No. 06-099A1; Test certificate No. D09-03.18 Rev.1–C of C No. R60/2000-CNI-00.01). The device was not recalibrated immediately prior to the present experiment; however, before data collection, correct functioning and measurement accuracy were independently verified using a certified reference mass to confirm correspondence with the expected load within the device resolution. This verification procedure ensured traceability without referencing the MuscleLab system, thereby avoiding circular validation.

For long-term stability, recalibration of the load cell is recommended annually or following transport, mechanical modification, or detection of signal drift, in accordance with manufacturer specifications.

For all systems, force values were derived from the same outcome definition, namely the peak value of the raw force–time trace obtained during each maximal voluntary contraction, using identical temporal markers across devices. This ensured that agreement analyses reflected true measurement concordance rather than differences in signal processing or outcome extraction.

ISOMETRO operates using proprietary software (PCE-FG v.2.14), which allows real-time visualization of force–time curves, repetition of trials if required, data storage, and export in spreadsheet format for subsequent analysis.

Although formal usability metrics were not collected, practical aspects of device setup and operation were monitored during testing. Average preparation time per participant, including height adjustment and angle verification, was approximately 5 min. Completing the full testing battery (four tests, two trials per test, including rest intervals) required approximately 70–75 min. Height adjustment was performed by loosening a single knob, repositioning the sled, and verifying joint angles using a goniometer (Baseline Stainless PSYMTEC, Madrid, Spain). Both operators and participants reported that the device was comfortable and easy to use. These qualitative observations suggest efficient setup and reproducible adjustment across participants and test configurations.

(b) Load Cell

A high-precision external load cell (MuscleLab–Ergotest, Langesund, Norway) was used to verify internal measurement consistency. The sensor has a measurement range of 0–5000 N, 14-bit resolution (approximately 38 g per bit), and factory calibration accuracy of ±0.1%. The load cell was connected in series with the ISOMETRO cable so that both devices were exposed to identical tensile loads during each trial. Data were sampled at 200 Hz, matching the acquisition frequency of ISOMETRO and the force plate systems, enabling synchronized signal comparison.

Because the load cell and ISOMETRO shared the same mechanical loading pathway, this comparison was used to verify signal consistency and measurement chain integrity rather than independent criterion validity.

The external load cell was used according to the manufacturer’s calibration specifications and was zeroed before each testing session. Annual recalibration is recommended by the manufacturer or after mechanical impact or transport.

(c) Force Plate

A force plate system (MuscleLab–Ergotest, Langesund, Norway) served as the independent criterion reference for vertical force measurement. The plate has a sensitivity of 0.01 N and a factory calibration error of <0.2%. During each test, participants maintained a stable stance on the plate, allowing simultaneous recording of the vertical ground reaction force generated during upper-limb tensile contractions.

The force plate was used according to the manufacturer’s factory calibration specifications and was zeroed prior to each testing session.

Force plate data were sampled at 200 Hz and time-synchronized with ISOMETRO and load cell recordings via a common MuscleLab hardware trigger system using MuscleLab acquisition software (Ergotest Technology AS, Langesund, Norway), which initiated simultaneous acquisition across the three devices. This hardware-based triggering ensured precise temporal alignment. No software-based post-alignment procedures were required beyond trimming the recordings to the synchronized acquisition window.

### 2.4. Procedures and Experimental Approach

**Figure 3:**
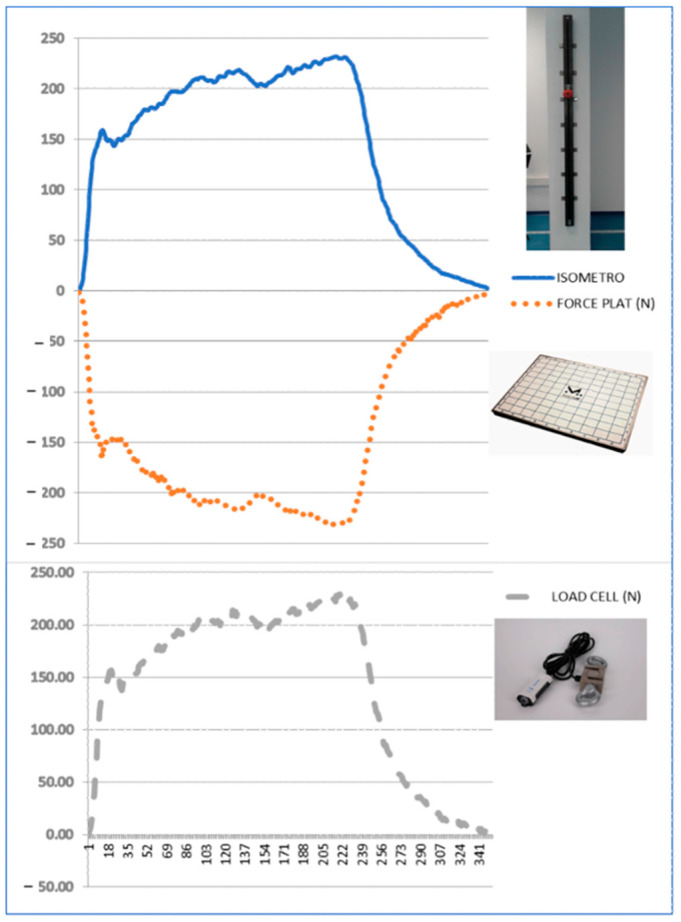
Representative example of synchronized force–time signals recorded simultaneously by ISOMETRO, the series-connected reference load cell, and the force plate during a single upper-limb tensile trial. The figure illustrates the temporal correspondence between the three systems and the opposite sign of the force plate signal, reflecting the vertical ground reaction force generated during downward traction. Peak force was extracted from the unfiltered raw signal for agreement analyses.

The force–time curve was recorded simultaneously using ISOMETRO, the series-connected reference load cell, and the force plate. All devices were sampled at 200 Hz and synchronized via a common hardware trigger system (MuscleLab) using MuscleLab acquisition software (Ergotest Technology AS, Langesund, Norway), allowing simultaneous acquisition of the same tensile action across three independent sensing systems. All tests were performed under vertical tensile conditions so that the applied load was aligned with the vertical axis of the three measurement systems and horizontal components were minimized. Participants generated a downward tensile force on the handle; by Newton’s third law, this produced an equal and opposite vertical ground reaction force measured by the force plate. A representative example of the synchronized force–time signals obtained from the three systems during one trial is shown in Figure 3.

The force plate served as the independent criterion device for concurrent validity because it provides an external measure of the vertical reaction force generated during traction. In addition, it enabled objective verification of force-vector alignment: the force plate records anteroposterior and mediolateral components, allowing detection of off-axis pulling. The ISOMETRO rail-guided sled mechanically constrained motion to the vertical plane (Z axis), reducing the likelihood of off-axis deviations. The force plate signals were used to confirm whether any non-vertical components occurred during each attempt. Any trial showing visible compensatory movements or detectable off-axis components on the force plate was immediately repeated until a valid attempt was obtained. Off-axis force was continuously monitored in real time using the anteroposterior and mediolateral channels of the force plate. Although no fixed percentage threshold relative to the vertical force component was pre-specified, trials were repeated when horizontal force components were clearly observable in the force traces and judged to be mechanically inconsistent with the intended vertical traction task. Alignment was visually monitored by two trained evaluators throughout each attempt. Only trials meeting these qualitative alignment criteria, with minimal horizontal force relative to the vertical component, were retained for analysis.

Three days before testing, each participant completed a familiarization session. Participants received standardized verbal instructions, practiced the technique for each test, and could ask questions about the protocol. Adequate familiarization was defined as the ability to reproduce the required posture, maintain the prescribed joint angles, and apply traction without compensatory movements. A second familiarization session was planned if needed, but all participants met the criteria after the first session. On the testing day, participants completed a standardized 15-min warm-up supervised by a researcher (light jogging and submaximal practice attempts of the test positions) to reduce injury risk and improve reproducibility.

Before each test, the evaluator instructed the participant’s stance on the force plate and the grip on the handle. The height of the ISOMETRO sliding sled was adjusted to the participant and the specific test configuration, and the target joint angle was verified using a goniometer. Because sled height determines joint configuration and contributes to force-vector alignment, this adjustment was performed before each condition. Alignment and posture were visually monitored throughout, and participants were asked to remain still until the start cue.

**Figure 4:**
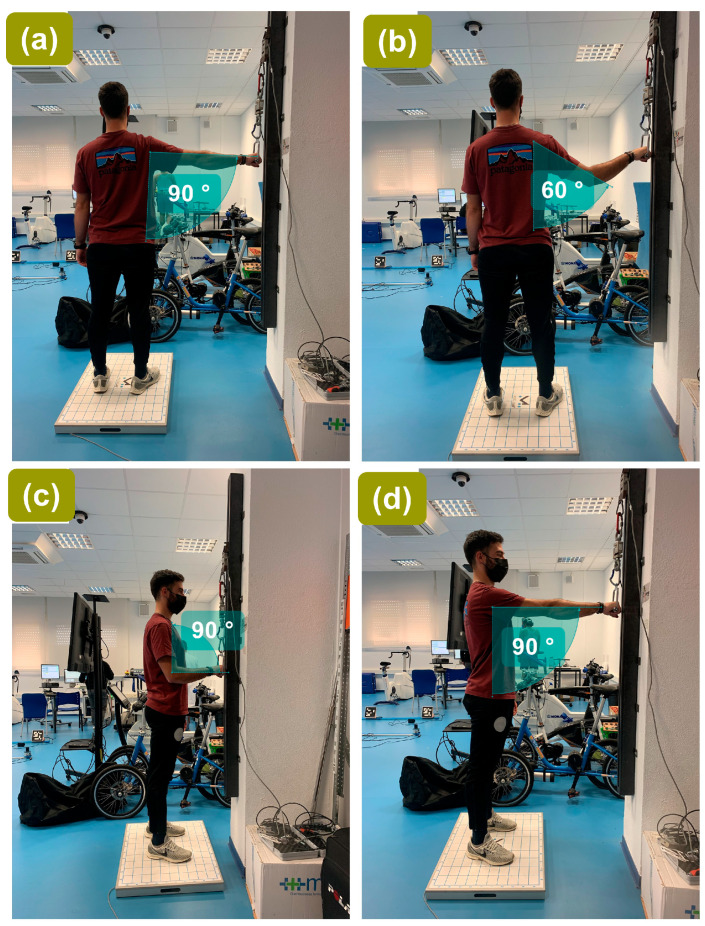
Upper-limb tensile isometric test configurations under vertical traction, with joint angles verified using a goniometer: (**a**) shoulder adduction at 90°; (**b**) shoulder adduction at 60°; (**c**) elbow extension at 90°; (**d**) shoulder extension at 90°. All tests were performed standing on the force plate with the traction line aligned vertically to minimize off-axis components.

Each participant performed four randomized upper-limb tensile tests: shoulder adduction at 90°, shoulder adduction at 60°, shoulder extension at 90°, and elbow extension at 90° (Figure 4). Test order and starting side were determined using a simple randomization procedure applied individually to each participant prior to testing, based on a computer-generated random sequence prepared before data collection. For each participant, the sequence of test conditions and the initial limb (right or left) were randomly assigned to minimize potential order and fatigue effects. For the shoulder adduction at 60° condition, the participant and handle position were adjusted to achieve the prescribed joint configuration while maintaining a vertical cable line. These joint positions were selected because they are commonly used in upper-limb isometric strength assessments and allow standardized testing with stable posture and minimized compensatory movement, consistent with previously reported protocols [39,40,41,42]. Two maximal trials were performed per condition on each side, alternating right and left hands, with 1 min rest between sides and 15 min rest between test conditions.

Each contraction began with a verbal cue (“start”). Participants were instructed to pull as fast and as hard as possible and to sustain maximal effort for at least 2 s. The evaluator provided standardized verbal encouragement and gave the cue “stop” once a clear decline in force was observed on the real-time force–time trace. Taring (zeroing) was performed before each attempt using the device software to reset the baseline to 0 N. Peak force was extracted as the primary outcome from the unfiltered raw signal for all devices, and only peak-force values were used for statistical analyses.

This study used an agreement-based validation framework with explicitly asymmetric evidential weight across the two reference comparisons. The force plate comparison provided independent concurrent validity for the vertical force component. The series-connected reference load cell was included as an internal consistency check of the shared measurement chain (handle → cable → sensing and acquisition pathway) under identical loading conditions; therefore, it does not provide independent validation of absolute force magnitude.

All tests were conducted in an exercise physiology laboratory under controlled environmental conditions (22 ± 1 °C; 50–60% relative humidity). Sessions were scheduled consistently between 10:00 a.m. and 12:00 p.m. to reduce potential circadian variability [43,44]. After testing, signals from all devices were exported for analysis, and force was expressed in newtons (N). Two maximal trials were collected for each condition and side to ensure correct execution and alignment; when both trials were valid, the highest peak-force value was retained as the representative measure for that condition and side. This approach is consistent with common practice in maximal voluntary contraction testing, where the highest reproducible peak value is considered to best represent maximal neuromuscular capacity and to minimize potential underestimation due to transient submaximal effort or fatigue [45]. This resulted in one peak-force value per participant, test, and side (21 participants × 4 tests × 2 sides = 168 observations) for the agreement analyses.

### 2.5. Statistical Analysis

Descriptive statistics are reported as mean ± standard deviation (SD). The primary objective of the statistical analysis was to evaluate measurement agreement between ISOMETRO and the force plate, which served as the independent reference criterion. Comparisons between ISOMETRO and the in-series load cell were conducted as secondary analyses to examine internal measurement consistency rather than independent validation.

Because measurements were nested within participants and repeated across tests and sides, linear mixed-effects models were used to account for the hierarchical data structure. For each comparison, alternative model specifications were explored, and the most parsimonious model was selected based on Akaike Information Criterion (AIC) and Bayesian Information Criterion (BIC). Final models included random intercepts for participants and fixed effects for test type when supported by model fit. Model assumptions were evaluated using residual diagnostics and formal tests (Shapiro–Wilk test for normality and Breusch–Pagan test for homoscedasticity).

Each participant contributed one representative peak-force value per side for each of the four upper-limb tests (21 participants × 4 tests × 2 sides = 168 observations). These observations reflect repeated measures within individuals and were not treated as independent samples. Two maximal trials were collected per condition and side for quality control; when both trials were valid, the highest peak value was retained. No averaging across sides was performed.

Agreement between devices was primarily assessed using Bland–Altman analysis to quantify systematic bias and limits of agreement (LOA), calculated as the mean difference ± 1.96 × SD of the differences. Proportional bias was examined by linear regression of the differences against the means, and heteroscedasticity was assessed using the Breusch–Pagan test. As complementary descriptors of agreement, Lin’s concordance correlation coefficient (CCC) and the intraclass correlation coefficient [ICC (2,1)] were computed with 95% confidence intervals.

Measurement error was further characterized using the standard error of the estimate (SEE) and the mean absolute error (MAE). SEE reflects the dispersion of residuals around the fitted regression line, whereas MAE represents the average absolute deviation between devices and provides a directly interpretable estimate of absolute disagreement. These metrics were interpreted descriptively in the context of laboratory-based isometric force measurements rather than as strict acceptability thresholds.

Exploratory subgroup analyses were conducted stratified by test type and by force magnitude (tertiles: low, medium, high) to evaluate the consistency of agreement across measurement conditions. Individual participant plots were inspected to identify potential outliers or systematic deviations. Statistical significance was set at α = 0.05.

All analyses were performed using R software (version 4.2.2, R Foundation for Statistical Computing, Vienna, Austria) with the following packages: lme4 and lmerTest for mixed-effects modeling, BlandAltmanLeh for agreement analyses, DescTools for CCC, irr for ICC, and ggplot2 for data visualization.

## 3. Results

### 3.1. Model Specification and Diagnostics

Alternative linear mixed-effects model specifications were compared using information criteria (Table S1) for both the primary (ISOMETRO vs. force plate) and secondary (ISOMETRO vs. in-series load cell) comparisons. The most parsimonious model that consistently provided the best fit included random intercepts for participants and test. This specification demonstrated improved fit relative to alternative models (ΔAIC = 5.4–8.2; ΔBIC = 4.9–7.6, depending on the comparison).

Model diagnostics confirmed that key assumptions were adequately met (see Supplementary Figures S1–S3). Residual-versus-fitted plots showed no systematic structure or curvature, supporting linearity and appropriate model specification. Visual inspection of Q–Q plots indicated approximate normality of residuals. The Shapiro–Wilk test showed W = 0.991 (*p* = 0.134) for the primary comparison and W = 0.989 (*p* = 0.098) for the secondary comparison, indicating no statistically significant deviation from normality. The Breusch–Pagan test for heteroscedasticity was non-significant across models (all *p* > 0.05), supporting homoscedasticity across the range of fitted values.

Variance partitioning (Table S2) indicated that between-participant variability accounted for approximately 45–49% of the total variance, with the remaining 51–55% attributable to within-participant (residual) variability across tests and sides under standardized laboratory conditions. No influential outliers were detected.

### 3.2. Overall Agreement Between Devices

**Figure 5:**
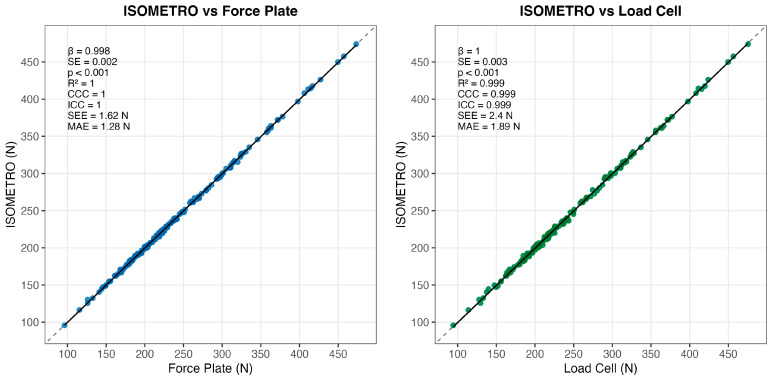
Linear regression analysis of ISOMETRO agreement with reference devices. Left panel: ISOMETRO vs. force plate. Right panel: ISOMETRO vs. load cell. Solid black lines represent simple linear regression models, and shaded areas indicate 95% confidence intervals for the fitted regression lines. The dashed diagonal line represents the line of identity (perfect agreement). Each panel displays the corresponding statistical parameters—slope (β), standard error (SE), *p*-value (p), coefficient of determination (R^2^), concordance correlation coefficient (CCC), intraclass correlation coefficient [ICC (2,1)], standard error of the estimate (SEE), and mean absolute error (MAE)—which summarize the agreement between ISOMETRO and each reference device. Regression lines are shown for visualization using ordinary least squares; mixed-effects model estimates are reported in Table 1.

In the primary criterion comparison, linear mixed-effects regression indicated near-unity agreement between ISOMETRO and force plate measurements (β = 0.995, SE = 0.003, *p* < 0.001), with a marginal R^2^ of 0.9998 (Table 1). In the secondary internal-consistency comparison, ISOMETRO also showed near-unity agreement with the in-series load cell (β = 0.998, SE = 0.003, *p* < 0.001; marginal R^2^ = 0.999). The corresponding linear regression analyses and graphical agreement patterns are shown in Figure 5.

**Figure 6:**
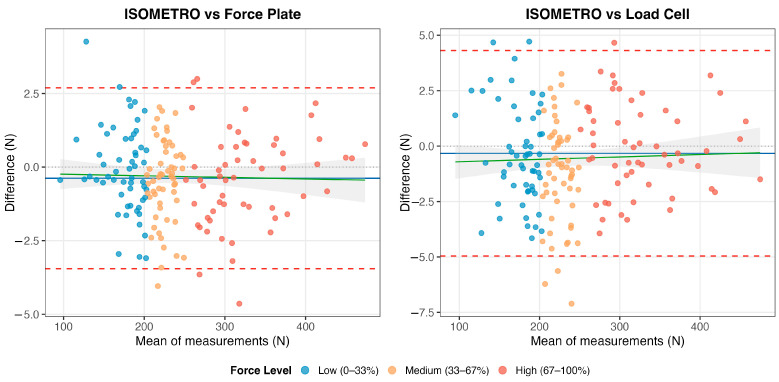
Bland–Altman plots for ISOMETRO agreement with force plate (**left**) and load cell (**right**). Blue line = mean bias; red dashed lines = 95% limits of agreement; green line = regression trend. Points colored by force level tertiles (blue = low, orange = medium, red = high). No evidence of proportional bias (*p* > 0.05).

Bland–Altman analysis showed minimal systematic bias for ISOMETRO relative to the force plate (bias = −0.38 N; 95% LOA: −3.45 to 2.69 N) and relative to the in-series load cell (bias = −0.33 N; 95% LOA: −4.96 to 4.31 N) (Figure 6). There was no evidence of proportional bias in either comparison based on regression of differences on means (force plate: *p* = 0.352; load cell: *p* = 0.734). Tests for heteroscedasticity were also non-significant (force plate: *p* = 0.841; load cell: *p* = 0.129), consistent with approximately constant variance across the observed force range.

Agreement indices were correspondingly high (Table 1). For the criterion comparison (ISOMETRO vs. force plate), CCC and ICC (2,1) were ≥0.999 with extremely narrow confidence intervals. For the internal-consistency comparison (ISOMETRO vs. load cell), both coefficients were 0.999. Error metrics were small: SEE was 1.62 N (force plate) and 2.40 N (load cell), and MAE was 1.28 N and 1.89 N, respectively.

For context, agreement between the two reference devices (load cell vs. force plate) was also near unity (β = 0.997, SE = 0.002, *p* < 0.001; bias = −0.06 N; Table 1), supporting the integrity of the shared measurement chain and the external criterion signal under the study configuration.

### 3.3. Agreement by Test Type

Agreement metrics were consistently high across the four upper-limb tests (Table 2). For the primary criterion comparison (ISOMETRO vs. force plate), CCC ranged from 0.999 to 1.000, and ICC (2,1) ranged from 0.999 to 1.000 across tests. Systematic bias remained small in all conditions (range: −0.96 to −0.07 N). Limits of agreement were similarly narrow, with LOA widths ranging from 5.84 N (elbow extension at 90°: −3.10 to 2.74 N) to 6.01 N (shoulder adduction at 90°: −3.96 to 2.05 N).

A comparable pattern was observed for the secondary internal-consistency comparison (ISOMETRO vs. in-series load cell), with CCC values between 0.997 and 1.000 and small biases across tests (Table 2), indicating consistent measurement behavior across test configurations under controlled vertical traction.

### 3.4. Agreement by Force Magnitude

Stratified analyses by force magnitude tertiles showed that agreement remained high across low, medium, and high force ranges (Table 3). For the primary criterion comparison (ISOMETRO vs. force plate), bias was small across tertiles (low: −0.19 N; medium: −0.44 N; high: −0.52 N), and LOA widths were similar (approximately 5.74 to 6.49 N across tertiles), consistent with the absence of heteroscedasticity in the overall analysis. Concordance remained high across tertiles (CCC and ICC: 0.996–0.999).

For the internal-consistency comparison (ISOMETRO vs. load cell), agreement also remained high across tertiles (CCC and ICC: 0.989–0.999), with slightly wider LOA in the lowest-force tertile (Table 3), suggesting that absolute dispersion may be more noticeable at lower force magnitudes even when relative concordance remains strong.

### 3.5. Individual Analysis

Participant-level inspection did not reveal systematic outliers or consistent deviations from agreement patterns (Figures S4 and S5). Across participants, observations clustered closely around the line of identity in regression plots and within narrow Bland–Altman limits, supporting robust criterion agreement under the tested laboratory configuration.

## 4. Discussion

The purpose of this study was to evaluate agreement between the ISOMETRO system and an independent criterion device (force plate) for upper-limb isometric tensile force measurements under a controlled laboratory configuration, while using a series-connected load cell as an internal consistency check of the measurement chain. The results demonstrated excellent agreement between ISOMETRO and the force plate, characterized by near-unity regression slopes, negligible systematic bias, narrow limits of agreement, and very high concordance indices. These findings indicate that, within the specific vertical traction configuration, sample characteristics, and standardized laboratory conditions tested, ISOMETRO provides force measurements that closely match those obtained from an established reference system. These results should therefore be interpreted strictly within this defined experimental context.

Importantly, the comparison with the in-series load cell should be interpreted as verification of internal measurement consistency rather than independent validation of absolute force magnitude. Because both sensors were exposed to the same mechanical loading pathway, their agreement primarily reflects the integrity and stability of the shared transmission and acquisition chain. In contrast, the force plate provides an external measurement of the vertical ground reaction force and therefore serves as the appropriate independent criterion for evaluating agreement in this configuration. The convergence of high agreement across both comparisons supports the external agreement of ISOMETRO under the tested configuration and confirms the internal stability of the measurement chain.

The near-unity regression slopes observed should also be interpreted in light of the strictly constrained vertical configuration employed in this study. Because all systems were mechanically aligned along the same loading axis and quantified the same vertical force component under highly standardized laboratory conditions, a strong linear correspondence and a high degree of statistical collinearity between devices were expected. Therefore, the regression coefficients close to unity should not be interpreted as evidence of perfect interchangeability across broader contexts, but rather as reflecting the controlled mechanical alignment and shared loading direction inherent to the experimental design.

It is important to acknowledge that when two sensors share an identical mechanical transmission pathway, high agreement does not necessarily preclude the presence of systematic errors affecting both systems simultaneously. Installation deviations, minor alignment imperfections, cable elasticity, or friction within mechanical components could theoretically influence the force transmitted through the shared pathway, thereby producing highly consistent yet proportionally biased measurements. For this reason, the series-connected load cell comparison cannot be interpreted as proof of absolute external accuracy. Rather, it serves to confirm internal measurement-chain stability under the tested configuration. The inclusion of the force plate as an independent external criterion device is therefore critical, as it allows evaluation of ISOMETRO’s agreement against a mechanically independent reference measurement.

The present findings are consistent with previous validation studies showing strong agreement between load cell–based systems and force plates when testing conditions are mechanically constrained and force vectors are well controlled [26,33,37,46]. Conversely, prior work has reported larger bias and variability when portable or examiner-stabilized devices are used under less constrained conditions [47]. In this context, the negligible bias observed in the present Bland–Altman analyses is likely attributable to the vertically guided configuration, which minimized off-axis loading and ensured uncontrolled transverse force components. The additional confirmation of agreement against an independent reference (force plate), rather than solely against a series-connected sensor, further supports that the observed agreement is not merely an artefact of shared mechanical coupling.

Several studies have highlighted that standardized alignment and mechanical guidance substantially improve measurement reproducibility and agreement by reducing horizontal and mediolateral force components and minimizing examiner-dependent stabilization [25,48,49]. ISOMETRO extends these principles by integrating a fixed vertical rail, an adjustable sled, and an embedded sensing pathway within a single wall-mounted system. This design facilitates repeatable joint positioning and a constrained line of pull across participants and test conditions, which likely contributed to the consistency observed in the present agreement analyses.

In contrast to handheld dynamometers, which depend heavily on examiner stabilization and are susceptible to counterforce limitations, joint torque leakage, and inter-rater variability, the rail–slider architecture of ISOMETRO mechanically constrains the line of pull to a single vertical axis. This reduces the influence of evaluator strength, minimizes unintended transverse force components, and limits variability introduced by manual positioning. Compared with wall-mounted or externally anchored load-cell systems lacking guided translation, the vertically guided rail and sled mechanism standardizes joint alignment and cable trajectory across participants and repetitions. By restricting motion to a controlled linear pathway and minimizing rotational degrees of freedom, the system reduces mechanical dispersion and potential off-axis torque generation. These structural characteristics likely contribute to the narrow limits of agreement and near-unity regression slopes observed in the present study.

From a practical perspective, ISOMETRO addresses several limitations associated with commonly used strength assessment tools. Compared with isokinetic dynamometers, it requires substantially less space, infrastructure, and setup time. In contrast to hand-held dynamometers, the wall-mounted guided structure eliminates the need for manual stabilization by the evaluator and reduces operator-dependent variability. Although the system is not portable in the traditional sense, its compact dimensions and relatively simple installation make it suitable for laboratory-based environments where standardized alignment and repeatability are priorities. These characteristics support its potential utility for controlled experimental testing rather than immediate deployment in clinical or field settings.

The present study is subject to several limitations that should be considered when interpreting the findings. First, agreement was evaluated exclusively for four upper-limb tensile configurations under vertical loading; therefore, the results cannot be generalized to other joint positions, force directions, or pulley-based configurations without further validation. Second, the sample consisted of young amateur rock climbers who were regularly physically active, limiting extrapolation to sedentary individuals, clinical populations, older adults, children, or elite athletes. Although the primary aim of this study was to evaluate mechanical agreement between measurement systems rather than to compare performance across populations, agreement properties may vary under different neuromuscular characteristics, motor control strategies, strength levels, or movement patterns. Therefore, the near-perfect concordance observed in the present study should not be assumed to apply to other populations or functional contexts without direct empirical verification. Third, all measurements were obtained under controlled laboratory conditions; performance under less controlled or field-based environments remains unknown. Fourth, the study was designed to assess agreement within a single session and did not evaluate test–retest reliability, inter-rater reliability, or responsiveness to change. Consequently, the present results do not support longitudinal monitoring or clinical decision-making applications. Fifth, only peak force was analyzed; temporal force characteristics such as rate of force development or force steadiness were not examined and warrant future investigation. Finally, although mechanical sources of error were minimized through careful setup and calibration, residual sources such as cable friction, guide-rail interaction, or minor alignment variability cannot be fully excluded. In addition, even under strictly vertical loading conditions, minute static friction at the rail–sled interface may be present. Although such friction is expected to be negligible relative to the high force magnitudes generated during maximal voluntary contractions in this study, it could introduce small measurement deviations at very low force levels. This potential effect should be considered in future studies examining submaximal or low-intensity force outputs or applications requiring high sensitivity at low loads.

Future research should extend agreement testing to additional joint configurations and force directions, assess reliability across repeated sessions and evaluators, and evaluate performance in more heterogeneous populations and less controlled environments. Incorporating temporal force metrics and examining sensitivity to meaningful physiological change will also be necessary before broader applied use can be recommended. Within its current scope, the present study demonstrates excellent agreement between ISOMETRO and an independent force plate reference for upper-limb tensile isometric measurements performed under a standardized vertical laboratory configuration.

## 5. Conclusions

This study demonstrates excellent agreement between the ISOMETRO system and an independent force plate reference for upper-limb isometric tensile force measurements performed under a standardized vertical traction configuration in a controlled laboratory environment. Near-unity regression slopes, negligible systematic bias, narrow limits of agreement, and very high concordance indices indicate that ISOMETRO provides peak force measurements that closely match those obtained from the criterion device within the specific testing conditions evaluated. Agreement with the in-series load cell further supports internal measurement-chain consistency but does not constitute independent validation of absolute force magnitude.

These conclusions are strictly limited to the experimental configuration examined, which included four upper-limb tensile tests (shoulder adduction at 90° and 60°, shoulder extension at 90°, and elbow extension at 90°), vertical loading alignment, young amateur rock climbers, and laboratory-controlled conditions. The study did not assess test–retest reliability, inter-rater reliability, responsiveness to change, alternative joint configurations, multidirectional force applications, or non-vertical pulley setups. Consequently, the present findings support the use of ISOMETRO for agreement-based laboratory assessment of peak upper-limb isometric tensile force under controlled conditions. However, extrapolation to other populations (e.g., clinical groups, older adults, sedentary individuals, or elite athletes), other force directions, or field-based environments requires further dedicated validation and reliability studies.

## 6. Patents

González-Montesinos, J.L.; España-Romero, V.; Fernández-Santos, J.R.; Jiménez-Pavón, D. System for the Evaluation and Training of Isometric Strength Using a Guiding System. Patent No. ES-2646730_B2, University of Cádiz, Spain, 2018.

## Figures and Tables

**Table 1:** Mixed-Effects Regression and Agreement Metrics.

Comparison	β (SE)	R^2^	Bias (N)	LOA (N)	CCC (95% CI)	ICC (95% CI)	SEE (N)	MAE (N)
ISOMETRO vs. Force Plate	0.995 (0.003)	0.9998	−0.38	−3.45 to 2.69	1.000 (1.000–1.000)	1.000 (1.000–1.000)	1.62	1.28
ISOMETRO vs. Load Cell	0.998 (0.003)	0.999	−0.33	−4.96 to 4.31	0.999 (0.999–1.000)	0.999 (0.999–1.000)	2.40	1.89
Load Cell vs. Force Plate *	0.997 (0.002)	0.999	−0.06	−3.55 to 3.44	1.000 (1.000–1.000)	1.000 (1.000–1.000)	1.79	1.40

β, regression slope; SE, standard error; R^2^, coefficient of determination; LOA, limits of agreement; CCC, concordance correlation coefficient; ICC, intraclass correlation coefficient [ICC (2,1)]; SEE, standard error of the estimate; MAE, mean absolute error. Regression slope (β) and associated inference were obtained from the linear mixed-effects model. * Agreement between the two reference devices (load cell and force plate) included for comparison. All *p*-values < 0.001 for regression slopes.

**Table 2:** Agreement Statistics by Test Type.

Comparison	Test	*n*	Mean Force (N)	SD (N)	Bias (N)	SD Diff	LOA Lower	LOA Upper	CCC	ICC
ISOMETRO vs. Force Plate	Elbow Extension at 90°	42	333	61.7	−0.18	1.49	−3.10	2.74	1.000	1.000
	Shoulder Adduction at 90°	42	240	47.1	−0.96	1.54	−3.96	2.05	0.999	0.999
	Shoulder Adduction at 60°	42	191	35.3	−0.32	1.68	−3.62	2.98	0.999	0.999
	Shoulder Extension at 90°	42	211	36.9	−0.07	1.46	−2.93	2.78	0.999	0.999
ISOMETRO vs. Load Cell	Elbow Extension at 90°	42	333	61.6	−0.01	1.73	−3.40	3.38	1.000	1.000
	Shoulder Adduction at 90°	42	239	46.9	−0.74	2.66	−5.96	4.48	0.998	0.998
	Shoulder Adduction at 60°	42	191	35.0	−0.22	2.20	−4.53	4.09	0.998	0.998
	Shoulder Extension at 90°	42	211	36.7	−0.34	2.75	−5.73	5.06	0.997	0.997

SD, standard deviation; LOA, limits of agreement; CCC, concordance correlation coefficient; ICC, intraclass correlation coefficient [ICC (2,1)]. Agreement metrics are presented separately for the primary criterion comparison (ISOMETRO vs. force plate) and the secondary internal-consistency comparison (ISOMETRO vs. in-series load cell) across four upper-limb isometric strength tests. All measurements are expressed in newtons (N).

**Table 3:** Agreement statistics stratified by force magnitude tertiles.

Comparison	Force Category	n	Mean Force (N)	Range (N)	Bias (N)	SD Diff	LOA Lower	LOA Upper	CCC	ICC
ISOMETRO vs. Force Plate	Low (0–33%)	56	174	128.4–201.5	−0.19	1.58	−3.29	2.91	0.996	0.996
	Medium (33–67%)	56	231	201.7–257.8	−0.44	1.46	−3.31	2.43	0.996	0.996
	High (67–100%)	56	326	257.9–461.9	−0.52	1.66	−3.77	2.72	0.999	0.999
ISOMETRO vs. Load Cell	Low (0–33%)	56	174	131.8–201.4	−0.35	2.74	−5.72	5.03	0.989	0.989
	Medium (33–67%)	56	231	201.6–257.0	−0.35	2.18	−4.61	3.92	0.991	0.991
	High (67–100%)	56	326	257.5–462.3	−0.28	2.18	−4.55	3.98	0.999	0.999

Force categories were defined using tertiles of the overall force distribution. SD, standard deviation; LOA, limits of agreement; CCC, concordance correlation coefficient; ICC, intraclass correlation coefficient [ICC (2,1)]. Agreement metrics are presented separately for the primary criterion comparison (ISOMETRO vs. force plate) and the secondary internal-consistency comparison (ISOMETRO vs. in-series load cell), stratified by force magnitude tertiles. All measurements are expressed in newtons (N).

## Data Availability

The data presented in this study are available on request from the corresponding author. The data are not publicly available due to ethical restrictions.

## Supplementary Materials

The following supporting information can be downloaded at: https://www.mdpi.com/article/10.3390/s26051504/s1, Table S1. Model Comparison Statistics for Linear Mixed-Effects Models. Table S2. Variance Components from Linear Mixed-Effects Models. Figure S1. Q–Q plot for model residuals. Quantile–quantile plot showing standardized residuals against theoretical normal quantiles. Most points closely follow the diagonal reference line, indicating approximate normality of residuals. Minor deviations are observed in the distribution tails. Although the Shapiro–Wilk test indicates statistical deviation from normality (W = 0.976, *p* = 0.005), visual inspection suggests no substantial departure likely to affect model validity, particularly given the large number of observations and the robustness of mixed-effects modeling. Figure S2. Residuals versus fitted values plot. Residuals plotted against fitted values to assess homoscedasticity and model specification. The blue smoothed line with 95% confidence interval (gray shaded area) shows no systematic pattern, and the horizontal red dashed line at zero indicates the expected mean. Random scatter around zero with no funnel shape supports homoscedasticity (Breusch–Pagan *p* = 0.759). Figure S3. Scale–location plot. Square root of standardized residuals plotted against fitted values to assess homogeneity of variance. The blue smoothed line with 95% confidence interval remains approximately horizontal across the range of fitted values, supporting constant variance across force magnitudes. No systematic increase in residual spread is observed at higher fitted values. Figure S4. Individual participant agreement between ISOMETRO and force plate measurements. Individual scatter plots for all 21 participants showing ISOMETRO measurements (y-axis) plotted against force plate measurements (x-axis). Each panel represents one participant (numbered 1–21), with the red dashed line indicating the line of identity (perfect agreement). Points represent the retained peak-force values for each test and side. Figure S5. Individual participant agreement between ISOMETRO and load cell measurements. Individual scatter plots for all 21 participants showing ISOMETRO measurements (y-axis) plotted against load cell measurements (x-axis). Each panel represents one participant (numbered 1–21), with the red dashed line indicating the line of identity (perfect agreement). Points represent the retained peak-force values for each test and side.

## Abbreviations

The following abbreviations are used in this manuscript:

CCC	Concordance Correlation Coefficient
ICC	Intraclass Correlation Coefficient
IF	Isometric Force
IS	Isometric Strength
ISOMETRO	Isometric Strength Measurement Device
LOA	Limits of agreement
MAE	Mean Absolute Error
OEPM	Spanish Patent and Trademark Office
R^2^	Coefficient of Determination
SD	Standard Deviation
SEE	Standard Error of the Estimate
