# Supplementary material for: Agreement-Based Validation of ISOMETRO for Upper-Limb Isometric Tension Measurements
Source: Sensors (Basel). 2026 Feb 27;26(5):1504. doi: 10.3390/s26051504 (PMC12986810; doi:10.3390/s26051504)
Supplement: Supplementary file 1 [file sensors-26-01504-s001.zip › Supplementary Files.pdf]

Supplementary Files

Table S1. Model Comparison Statistics for Linear Mixed-Effects Models.

Comparison	Model	AIC	ΔAIC	BIC	ΔBIC	df
ISOMETRO vs Force Plate	Model 1 (RI: P + T)	385.2	0.0	398.4	0.0	5
	Model 2 (RI: P)	393.5	8.3	404.5	6.1	4
	Model 3 (RI: P, FE: T)	391.8	6.6	405.3	6.9	5
	Model 4 (FE only)	452.1	66.9	460.9	62.5	3
	Model 5 (RI: T)	445.7	60.5	456.7	58.3	4
ISOMETRO vs Load Cell	Model 1 (RI: P + T)	412.8	0.0	426.0	0.0	5
	Model 2 (RI: P)	419.3	6.5	430.3	4.3	4
	Model 3 (RI: P, FE: T)	418.1	5.3	431.6	5.6	5
	Model 4 (FE only)	478.5	65.7	487.3	61.3	3
	Model 5 (RI: T)	472.9	60.1	483.9	57.9	4

AIC, Akaike information criterion; BIC, Bayesian information criterion; df, degrees of freedom; RI, random intercept; P, participant; T, test; FE, fixed effect.
Model 1 includes random intercepts for both participant and test. Model 2 includes a random intercept for participant only. Model 3 includes a random intercept for participant with test as a fixed effect. Model 4 includes fixed effects only (no random effects). Model 5 includes a random intercept for test only.
Lower AIC/BIC values indicate better model fit. ΔAIC and ΔBIC represent differences from the best model (Model 1).
Model 1 was retained for all subsequent analyses.

Table S2. Variance Components from Linear Mixed-Effects Models.

Comparison	Source	Variance	SD	Proportion (%)
ISOMETRO vs Force Plate	Participant	2.85	1.69	45.2
	Test	0.12	0.35	1.9
	Residual	3.33	1.82	52.9
ISOMETRO vs Load Cell	Participant	4.12	2.03	48.7
	Test	0.08	0.28	0.9
	Residual	4.26	2.06	50.4

SD, standard deviation.
Variance components are derived from the best-fitting linear mixed-effects model (Model 1) with random intercepts for participant and test.

Proportion represents the percentage of total variance attributable to each component. All values are expressed in Newtons (N) or N^2.

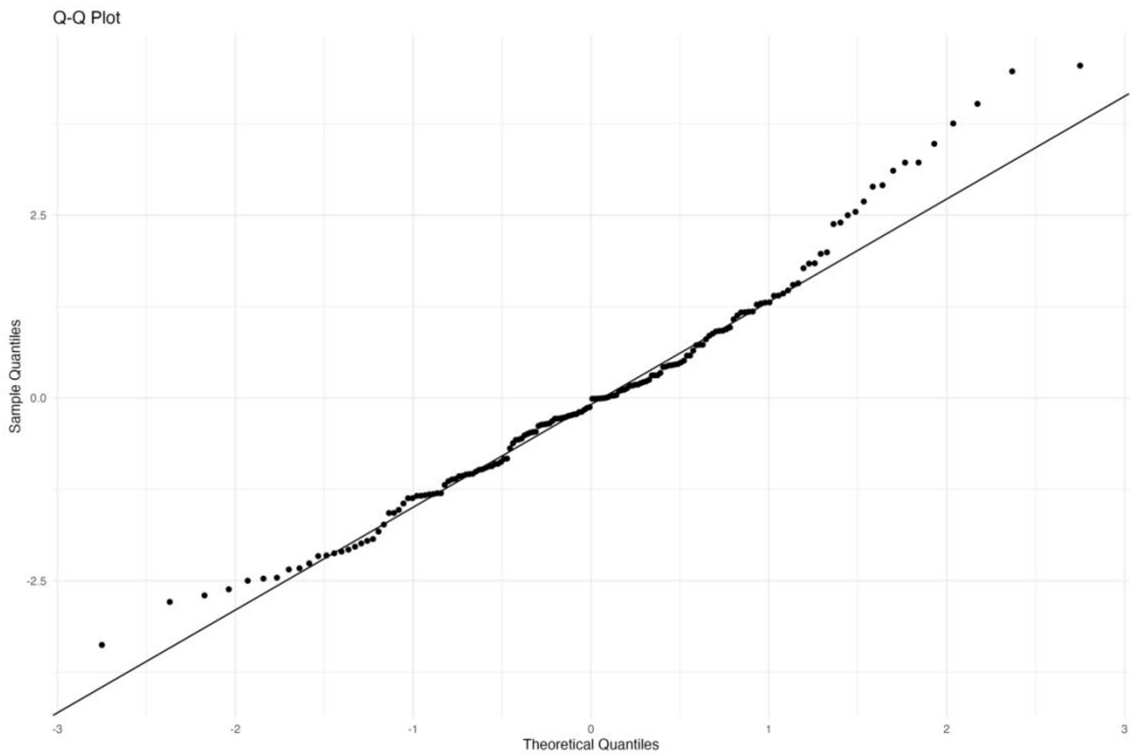

Figure S1. Q–Q plot for model residuals. Quantile–quantile plot showing standardized residuals against theoretical normal quantiles. Most points closely follow the diagonal reference line, indicating approximate normality of residuals. Minor deviations are observed in the distribution tails. Although the Shapiro–Wilk test indicates statistical deviation from normality ($W = 0.976$, $p = 0.005$), visual inspection suggests no substantial departure likely to affect model validity, particularly given the large number of observations and the robustness of mixed-effects modeling.

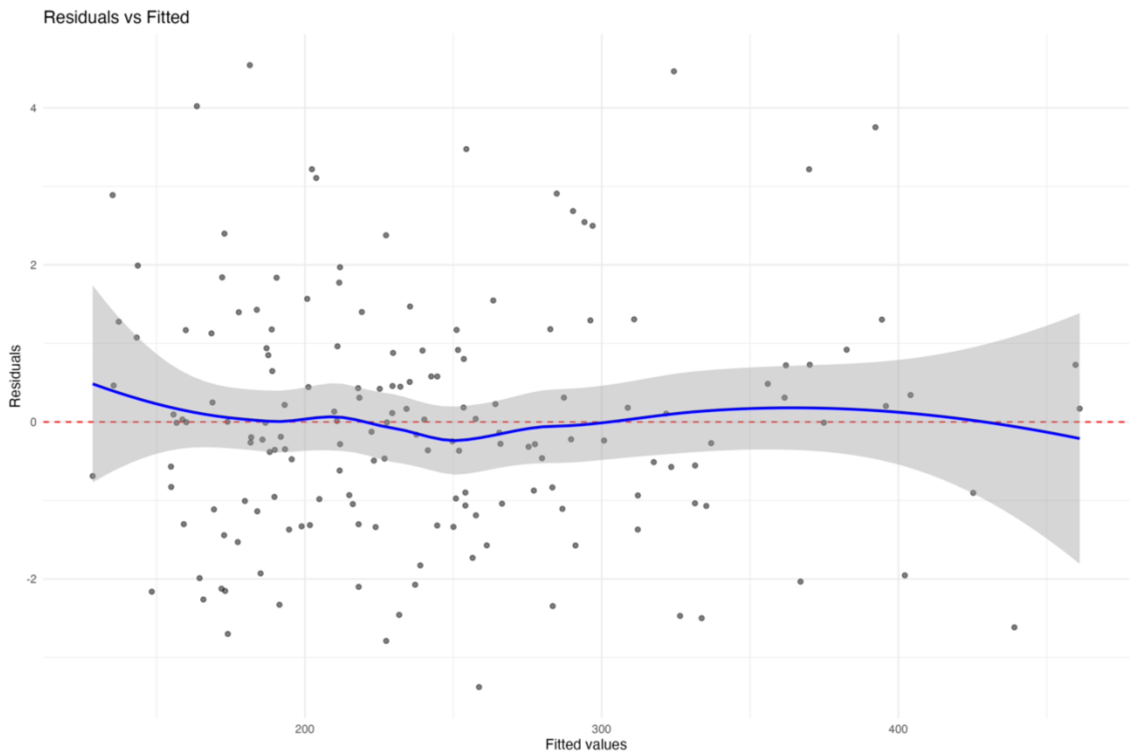

Figure S2. Residuals versus fitted values plot. Residuals plotted against fitted values to assess homoscedasticity and model specification. The blue smoothed line with 95% confidence interval (gray shaded area) shows no systematic pattern, and the horizontal red dashed line at zero indicates the expected mean. Random scatter around zero with no funnel shape supports homoscedasticity (Breusch–Pagan $p = 0.759$).

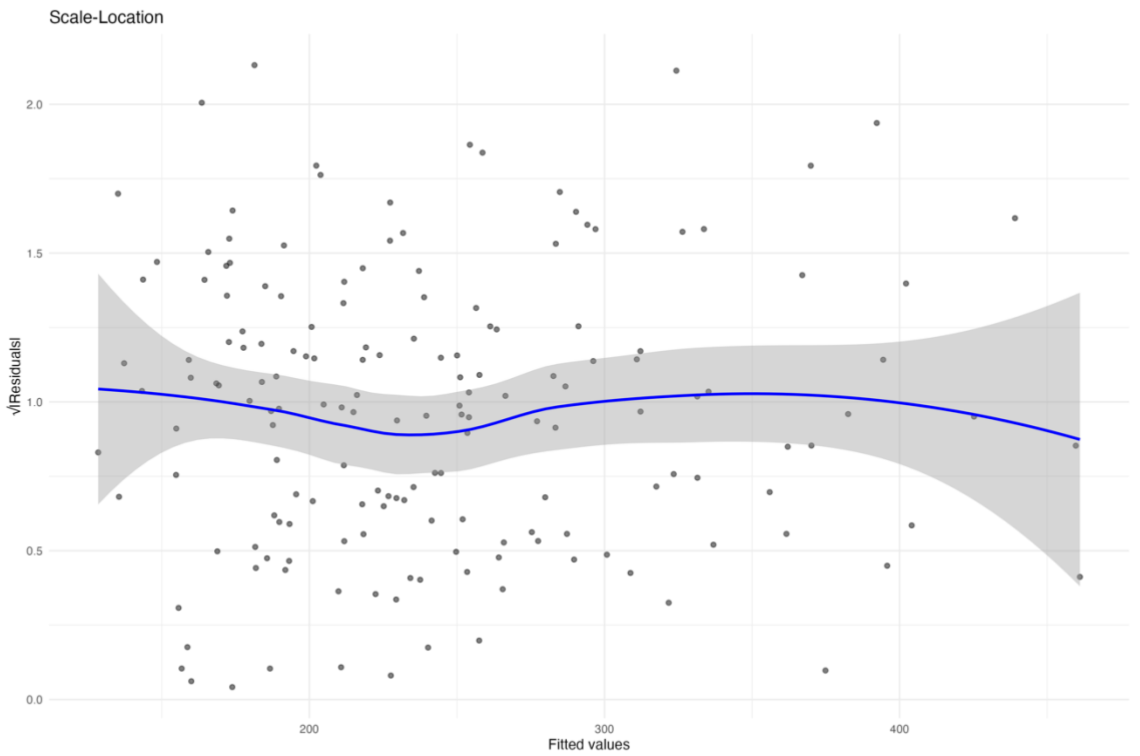

Figure S3. Scale–location plot. Square root of standardized residuals plotted against fitted values to assess homogeneity of variance. The blue smoothed line with 95% confidence interval remains approximately horizontal across the range of fitted values, supporting constant variance across force magnitudes. No systematic increase in residual spread is observed at higher fitted values.

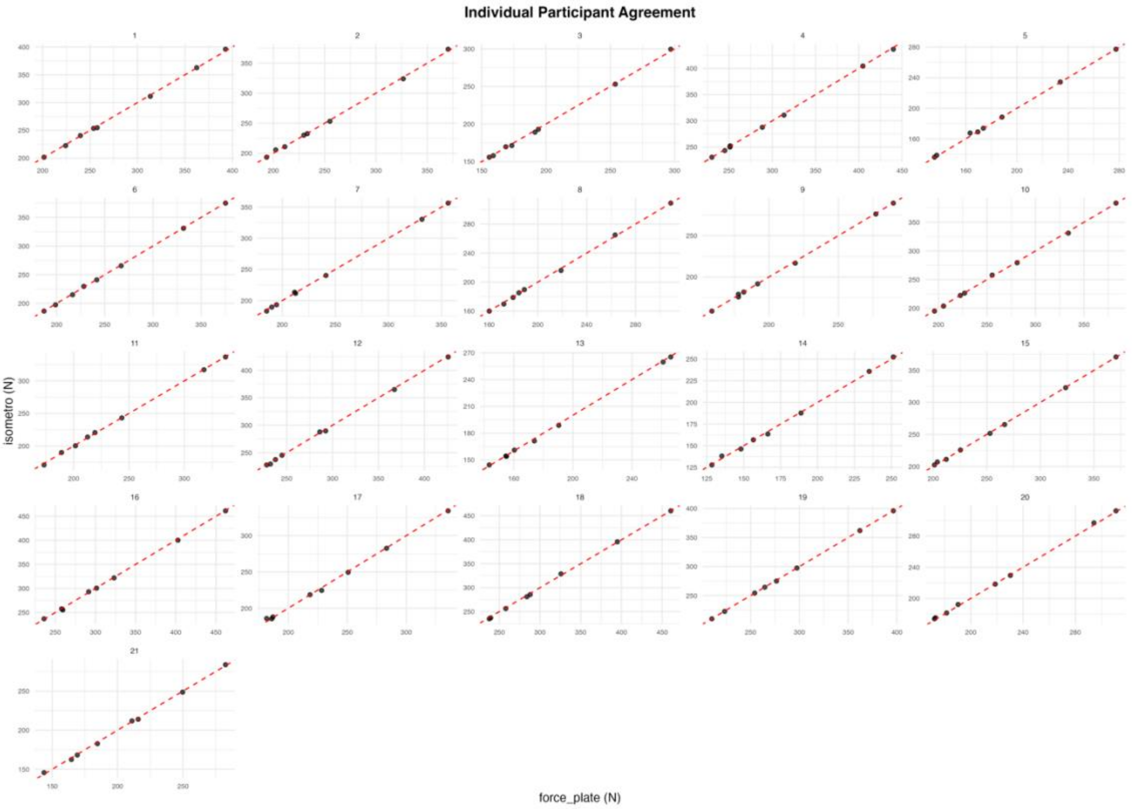

Figure S4. Individual participant agreement between ISOMETRO and force plate measurements. Individual scatter plots for all 21 participants showing ISOMETRO measurements (y-axis) plotted against force plate measurements (x-axis). Each panel represents one participant (numbered 1–21), with the red dashed line indicating the line of identity (perfect agreement). Points represent the retained peak-force values for each test and side.

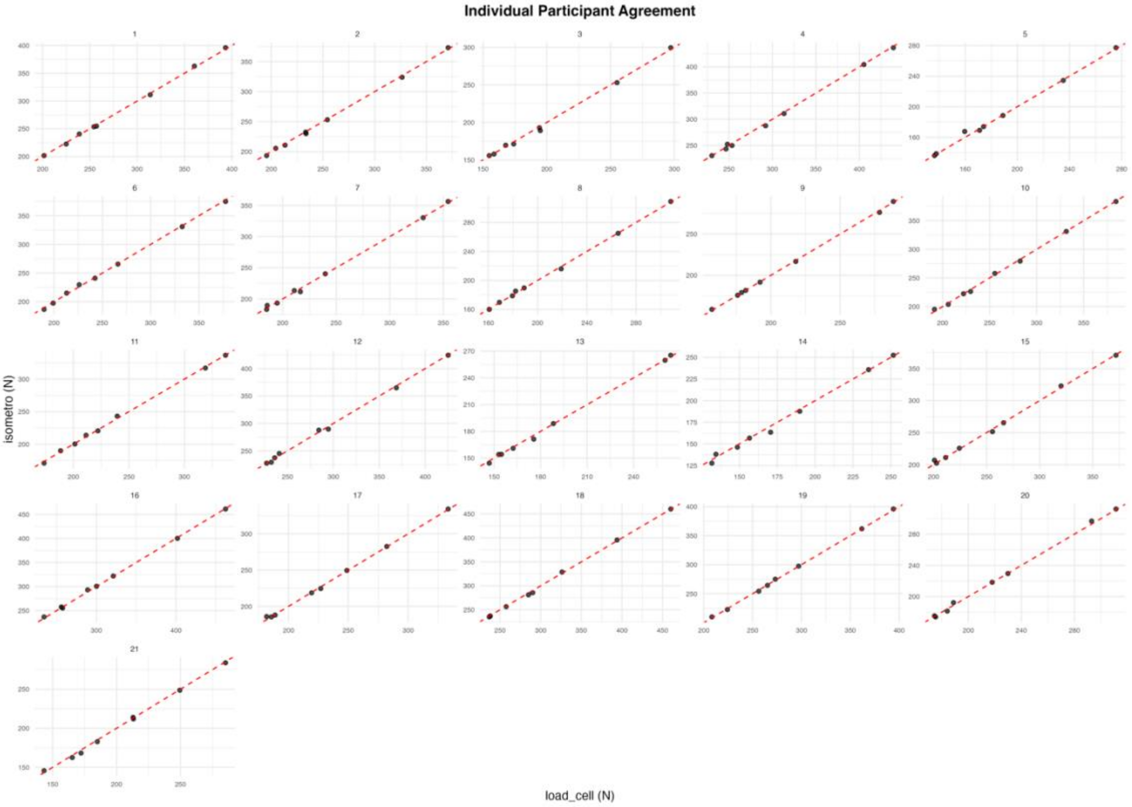

Figure S5. Individual participant agreement between ISOMETRO and load cell measurements. Individual scatter plots for all 21 participants showing ISOMETRO measurements (y-axis) plotted against load cell measurements (x-axis). Each panel represents one participant (numbered 1–21), with the red dashed line indicating the line of identity (perfect agreement). Points represent the retained peak-force values for each test and side.
